# A New Method for the Measurement of International Normalized Ratio in Hemodialysis Patients with Heparin-Locked Tunneled Dialysis Catheters

**DOI:** 10.1155/2020/7586437

**Published:** 2020-12-22

**Authors:** Céline B. Seghers, Kristien Ver Elst, Jolien Claessens, Steven Weekx, Sigrid Vermeiren, Manu Henckes

**Affiliations:** ^1^Department of Nephrology, GZA Ziekenhuizen, Campus Sint-Augustinus, Oosterveldlaan 24, Wilrijk 2610, Belgium; ^2^Department of Laboratory Medicine, GZA Ziekenhuizen, Campus Sint-Augustinus, Oosterveldlaan 24, Wilrijk 2610, Belgium

## Abstract

**Background:**

To measure International Normalized Ratio (INR) in hemodialysis patients with tunneled dialysis catheters (TDCs), blood sampling is frequently obtained via the catheter at the start of the session. INR measurements via finger-prick point of care testing (POCT) and via blood sampling taken from the dialysis circuit are evaluated as alternatives.

**Methods:**

In 14 hemodialysis patients with TDCs, treated with vitamin K antagonists (VKA), INR measurements via POCT were compared with plasma INR samples taken via the catheter at the start of dialysis and via the dialysis circuit after 30 and 60 minutes during 3 nonconsecutive dialysis sessions.

**Results:**

Blood samples taken at the start of dialysis at the catheter site were frequently contaminated with heparin originating from the locking solution (unfractionated heparin concentration (UFH) >1.0 IU/ml in 13.2%). POCT INR at the start of dialysis was not different from plasma INR after 30 and 60 minutes (Wilcoxon test *p*=0.113, *n* = 37, and *p*=0.631, *n* = 36, respectively). Moreover, there was no difference between POCT INR at the start of dialysis and POCT INR after 30 and 60 minutes (Wilcoxon test *p*=0.797 and *p* = 0.801, respectively; *n* = 36). Passing and Bablok regression equation was used, *y* = 0.460 + 0.733x; *n* = 105. Treatment decisions based on these 2 methods showed a very good overall agreement (kappa = 0.810; 95% CI: 0.732–0.889; *n* = 105).

**Conclusions:**

Measuring plasma INR via the TDC at the start of dialysis should be abandoned. Measuring POCT INR via a finger prick at the start or even after 30 to 60 minutes is an alternative. The most elegant alternative is to take plasma INR samples via the dialysis circuit 30 minutes or later after the start of the dialysis.

## 1. Introduction

Many hemodialysis patients are treated with vitamin K antagonists (VKA) such as warfarin, fenprocoumon, and acenocoumarol, mostly to prevent cerebrovascular emboli in patients with atrial fibrillation. This indication is evidence based in high-risk patients with normal kidney function but is controversial in dialysis patients [1]. In these patients, the risk of emboligenic phenomena and also the risk of bleeding due to VKA therapy are importantly increased [2–6]. Moreover, in the study of Yang et al. [7], it is shown that hemodialysis patients have substantially reduced time inside INR therapeutic range. However, in concordance with the actual guidelines [8], most dialysis centres still use VKA but with very strict and frequent monitoring of the International Normalized Ratio (INR). Mostly, prothrombin time (PT) and INR are monitored at least once a week. In patients with tunneled dialysis catheters (TDCs), this is done via blood sampling at the start of dialysis via the catheter after removing the lock and flushing thoroughly.

According to the literature [9] and the clinical experience in our centre, contamination of the blood sample with the locking solution remains a frequent problem and leads to false high INR values.

For this reason, we decided to investigate other methods of measuring INR in dialysis patients with TDCs on VKA: point of care testing (POCT) INR on a blood droplet obtained via a finger prick at the start and 30 and 60 minutes after the start of dialysis and blood sampling for INR taken via the dialysis circuit 30 and 60 minutes after the start of dialysis.

## 2. Materials and Methods

All chronic dialysis patients with a TDC treated with VKA at the dialysis centre of GZA Ziekenhuizen, campus Sint-Augustinus (Antwerp, Belgium), were approached to participate in the study. The study was approved by the local Committee of Medical Ethics.

After informed consent, blood was taken for INR and PT measurement during 3 different (nonconsecutive) dialysis sessions for each patient. One blood sample was taken at the start of the dialysis session (*t*_0_), and one 30 minutes (*t*_30_) and one 60 (*t*_60_) minutes after the start. The first blood sample was obtained via the TDC after removing the locking solution and flushing thoroughly following a standardized procedure: the first 5 ml of aspirated catheter locking solution and blood are discarded; then, 10 ml of saline is injected through the catheter with another syringe, and then with this syringe, 10 ml of blood is aspirated from and immediately reinjected in the catheter for 5 times; then, the blood sample is taken. The samples at 30 and 60 minutes were obtained via the “arterial” sample port of the dialysis circuit. This is the sampling port that is located before blood passes the heparin pump and hemodialysis filter. Simultaneously, a POCT INR with a finger prick was obtained (at the start, and 30 and 60 minutes after the start of dialysis). All POCT INR values were measured by the same person on the same Coaguchek® XS Pro analyser (Roche Diagnostics, Switserland). CoaguChek® XS PT Test strip reagent uses an antiheparin agent (not specified) to neutralize UFH up to 0.8 IU/ml (package insert 201507V5.0). Before use, performance characteristics were approved by an internal validation protocol and by the participation to an external quality control (EQC) programme (ECAT, Leiden). POCT INR EQC results met the ECAT acceptance criteria (+/−15% deviation compared to the target INR).

Blood for plasma INR measurement was drawn in a Vacutainer^©^ tube, 2.7 ml, 0.109 M Na_3_ citrate (Beckton Dickinson, USA), and was analyzed later in the central laboratory of the hospital. The citrated samples were centrifuged (2000 g), and plasma was aliquoted and stored at −80°C within two hours after sampling. All INR analyses were measured in a single batch run after maximum 122 days of storage on a ACL TOP 500 analyser using HemosIL® ReadiPlasTin (Werfen, Spain). This reagent uses polybrene to neutralize UFH in plasma up to 1.0 IU/ml (package insert 03/2016). UFH concentrations were measured using HemosIL® Liquid anti-Xa (Werfen, Spain) with a lower limit of detection of 0,04 IU/ml.

As all paired data were not normally distributed, paired results were compared by a Wilcoxon test. Method comparison was performed by Passing and Bablok regression analysis.

Clinical agreement was determined using inter-rater agreement (kappa) statistics.

Statistical analysis was performed using MedCalc version 19.2.3 (MedCalc Software, Belgium)

## 3. Results

In 12 out of the 14 patients included in this study, we obtained 9 blood samples simultaneously with a POCT INR (3 dialysis sessions with sample taking at the start and after 30 and 60 minutes). Unfortunately, 2 patients expired during the study. In these patients, it was only possible to obtain blood samples and POCT INR during one dialysis session (for each patient 3 blood samples).

As a result 114 pairs of simultaneous plasma INR (via the dialysis catheter or circuit) and POCT INR (via finger prick), measurements on 3 different moments during the dialysis session (38 pairs at 3 different time points) were obtained. In one set of plasma INR measurements from the same dialysis session, the samples after 30 and 60 minutes show an extremely high UFH concentration due to incorrect sampling at the venous instead of arterial side of the dialysis circuit. Furthermore, one POCT INR measurement at 60 minutes is not correct because of partial coagulation of the droplet on the test strip. These results are excluded from the data set. The clinical data of the patients participating in the study are summarized in Table 1.

**Figure 1:**
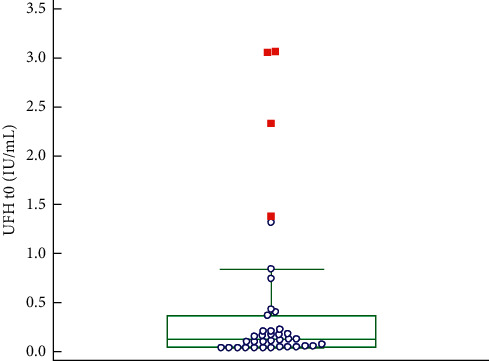
Box and Whisker plot UFH concentrations at the start; red squares represent far out values (>upper quartile +3 times the interquartile range).

The UFH concentrations obtained via the TDC at the start of dialysis are shown in Figure 1. The median UFH concentration is 0.128 IU/ml. In 5/38 samples (13.2%), the UFH concentration is above 1.00 IU/ml, despite that the strict flushing protocol was used. When UFH concentration in plasma is above 1.00 IU/ml, the neutralization of heparin by the test reagent will be incomplete, leading to incorrect PT and INR results.

The median concentration of UFH in the blood samples obtained via the dialysis circuit at the start and 30 and 60 minutes after the start of the dialysis is shown in Table 2, together with the POCT INR and plasma INR values at these different time points.

**Figure 2:**
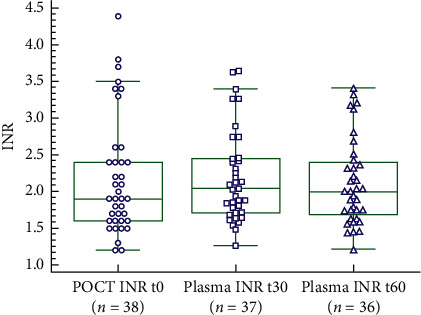
Box and Whisker plot POCT INR *t*_0_ and plasma INR *t*_30_ and *t*_60_.

The POCT INR measurements at the start of dialysis were compared with the plasma INR measurements via the dialysis circuit after 30 and 60 minutes (Figure 2).

When comparing the 30-minute plasma INR with the POCT INR measured at *t*_0_, no statistically significant difference is seen using Wilcoxon testing (*n* = 37, *p*=0.113). Similarly, no statistically significant difference is seen between POCT INR at the start of dialysis and plasma INR after 60 minutes (*n* = 36, *p*=0.631).

In 84.9% (62/73 samples), there is an acceptable difference of 15% or less between POCT and plasma INR (ECAT criteria).

Next, POCT INR at the start of dialysis was compared with POCT INR measurements at 30 and 60 minutes. Wilcoxon testing shows no significant difference (*n* = 37, *p*=0.797) between POCT INR measured at the start of dialysis (median 1.90, 95% CI: 1.70–2.38) and after 30 minutes (median 2.00, 95% CI: 1.80–2.48). The same analysis was performed comparing POCT INR at the start and 60 minutes after the start of dialysis (*n* = 36). Again no significant difference is seen (*p*=0.801, median POCT INR start 1.95, 95% CI: 1.70–2.40, median POCT INR 60 minutes 2.10, 95% CI: 1.70–2.43).

**Figure 3:**
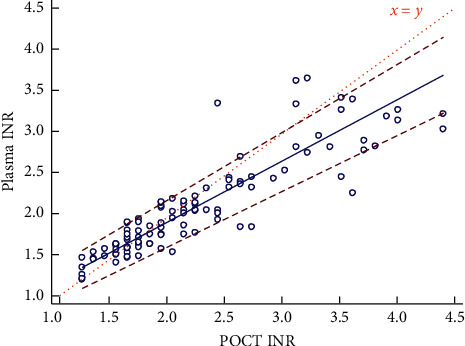
POCT and plasma INR comparisons in Passing and Bablok regression (*n* = 105). Regression equation: plasma INR = 0.46 (95% CI: 0.29–0.58) + 0.73 (95% CI: 0.66–0.81) of POCT INR.

A Passing and Bablok regression was made comparing plasma INR measurements with POCT INR measurement at the same time. The results are visualized in Figure 3. The relation between POCT and plasma INR was not statistically different from linearity.

Finally, the POCT and plasma INR results were compared based on treatment decisions. The most straightforward way to evaluate the treatment decision is to look whether the result is in the therapeutic (INR 23), subtherapeutic (INR < 2), or supratherapeutic range (INR > 3). In Table 3, all the POCT INR values were compared with the plasma INR values taken at the same moment.

Inter-rater statistics showed a kappa score of 0.810 (95% CI: 0.732–0.889). This corresponds to a very good agreement strength. Since there were no cases with a POCT INR > 3 and a plasma INR < 2 or with a POCT INR < 2 and a plasma INR > 3, no opposite treatment decisions would have been induced in clinical practice (dose increase versus dose decrease).

## 4. Discussion and Conclusion

In many hemodialysis centres, the common method of measuring prothrombin time and INR in patients with TDCs on VKA antagonists is via blood sampling at the start of dialysis via the catheter, after removing the lock and flushing thoroughly. This method has been seriously questioned by some investigators [9–11]. Rioux et al. [10] found in a small pilot study a falsely elevated INR measurement via blood sampling via the catheter in 56% ± 28% when compared to INR measurement via venipuncture before the start of the dialysis, which suggests that this is due to contamination with UFH from the locking solution. In our study, we could demonstrate these unacceptable high concentrations of UFH in 13.2% of the blood samples taken via the dialysis catheter at the start even with special emphasis on a strict flushing protocol. This confirms that plasma INR measurements should not be performed via blood samples taken from the dialysis catheter at the start of the dialysis in patients with a heparin lock, provided that there would be a good and practical alternative.

The most straightforward alternative would be a venipuncture at the start of dialysis to measure plasma INR. However, this seems unattainable because of the poor vascular health of the patients and the burden of an extra venipuncture at least once a week.

Another alternative is POCT INR measurement via a finger prick, a method of evaluation of INR which has been found equivalent to plasma INR measurements in other studies [9, 12, 13]. The POCT technique used in this study buffers UFH up to 0.8 IU/ml. Using this test, we found that timing of POCT INR at the start or after 30 and 60 minutes had no influence on the result despite the use of a moderate dose of UFH as an anticoagulant during dialysis (mean bolus dose of 31.9 IU/kg body weight, mean continuous dose over the 4 hours of dialysis of 22.5 IU/kg body weight). This means that POCT INR measurements can be done with a prompt result at a moment the workload for the dialysis nurse is less than at the start of the dialysis. However, a weekly finger prick to perform the POCT testing is in our experience not evident because of poor vascular status, calluses at the finger tips due to frequent glycaemia monitoring in many patients, and relative vasoconstriction during dialysis. Moreover, a lot of patients experience the finger prick as painful and annoying.

The next and by far most convenient alternative for the patient is a plasma INR measurement via the dialysis circuit at the arterial sampling port during the dialysis session. Rioux et al. described this novel technique of plasma INR measurements, taken via the arterial bloodline sample port after 1 h of dialysis and compared this with blood obtained via venipuncture prior to the start of the dialysis [14]. They found a minimally overestimated INR value (INR difference 0.2 ± 0.2) that was not considered clinically significant. However, they did not measure UFH concentrations in their study, nor did they mention whether the INR testing used an inherent buffer for UFH presence in the sample.

To the best of our knowledge, this is the first study that measured the UFH plasma concentrations using an anti-Xa assay at the start and after 30 and 60 minutes after the start of the dialysis, and that evaluated the effects of these UFH concentrations on the POCT INR and plasma INR measurements in hemodialysis patients with TDCs. We used INR testing that neutralizes UFH up to a concentration of 0.8 IU/ml with the CoaguChek® XS (for POCT INR) and up to 1.0 IU/ml with the HemosIL® ReadiPlasTin (plasma INR) technique. Our data show that, with the moderate dosing schedule of UFH that was used, the levels of UFH were always far below 1.00 IU/ml 30 and 60 minutes after the start of the dialysis which should lead to complete neutralization of UFH by the reagent. In accordance with this, our data show that these plasma INR measurements 30 and 60 minutes after the start of dialysis were not statistically different from POCT INR measurement at the start of dialysis, which could be considered as the reference value to compare with.

Also important is that we found a very good inter-rater agreement in the treatment decisions based on the POCT INR or on the plasma INR technique with a kappa score of 0.810 (95% CI: 0.732–0.889). Both techniques did not lead to opposite treatment decisions. In 80% of the measurements (84 out of 105 measurements), no change or a change in the same direction to reach the therapeutic range (INR 2.0–3.0) was recommended. In the remaining 21 out of the 105 measurements (20%), a treatment decision in one direction was proposed by only one of the measurements. Hoel et al. [9], who compared POCT INR and plasma INR, came to the same conclusion, with both INR measurements in agreement by  ±0.2 INR in 67% and by  ±0.4 INR in 89.2% of the time. They found the same treatment decision in 82%, which is in perfect agreement with our results.

Using citrate instead of heparin locks in patients on VKA would be another alternative technique, which we did not investigate. Although the effect of citrate on INR measurements is probably minimal [10], the disadvantages of this method should be taken into account [11]. A systematic review and meta-analysis concluded that benefits and harms of citrate versus heparin locking solutions remain unclear [15]. We are also aware that many dialysis centres use low-molecular weight heparins instead of UFH as anticoagulant during dialysis in these patients. The results of our study cannot be extrapolated to this other clinical setting.

In conclusion, this study suggests that measuring INR via a plasma sample taken 30 minutes or later after the start of the dialysis session is the best method of monitoring INR in patients with TDC. The plasma INR measurement is well tolerated, less expensive than POCT INR measurement, and easily applicable in the routine of the dialysis nurse because of the variable time frame in which the sample can be taken.

## Figures and Tables

**Table 1:** Baseline clinical characteristics.

Median age	81 years (minimum 70, maximum 88)
Sex	9 men
	5 women

Indication oral anticoagulant	Atrial fibrillation (*n* = 12)
	Thrombosis dialysis catheter (*n* = 1)
	Arterial embolisms (*n* = 1)

VKA	Warfarin (*n* = 12)
	Fenprocoumon (*n* = 2)

Target INR	INR 2–3 (*n* = 14)

Catheter	Arrow cannon II plus catheter (teleflex medical) 23 cm (*n* = 9)
	BioFlo^TM^ DuraMax (AngioDynamics Navilyst Medical) (*n* =2)
	Others (*n* = 3)
	All dual lumen catheters

Catheter lock	Heparin natrium (5000 IU/ml, *n* = 11) (B. Braun)
	Taurolock-Hep500^TM^ (contains 500 IU/ml UFH and citrate 4%) (*n* = 2) (TauroPharm GmbH)
	Citra-Lock^TM^ (citrate 4%, *n* = 1) (Dirinco N.V.)

Dialysis	Hemodialysis (*n* = 4)
	Hemodiafiltration predilution (*n* = 1)
	Hemodiafiltration postdilution (*n* = 5)
	Hemodiafiltration mixed (*n* = 4)
	All via Fresenius 5008 or 5008 S monitors

Dialysis filter	FX CorDiax 80^TM^ (Fresenius) (*n* =2)
	FX CorDiax 1000^TM^ (Fresenius) (*n* = 9)
	Nephral 400 ST^TM^ (Baxter) (*n* = 2)
	Polyflux 170 H ^TM^ (Gambro) (*n* = 1)

Anticoagulation during dialysis	Unfractionated heparin (UFH) (*n* = 14)
Mean heparin dose at start (in IV bolus)	31.9 IU/kg
Mean heparin dose during dialysis (in continuous infusion)	22.5 IU/kg

**Table 2:** Median UFH concentration, POCT INR, and plasma INR at *t*_0_, *t*_30_, and *t*_60_ with minimum and maximum (between brackets).

	*t*_0_ (*n* = 38)	*t*_30_ (*n* = 37)	*t*_60_ (*n* = 36)
UFH (IU/ml)	0.128 (<0.04–3.064)	0.418 (<0.04–0.864)	0.340 (<0.04–0.712)
POCT INR	1.9 (1.2–4.4)	2.0 (1.2–4.0)	2.1 (1.2–4.4)
Plasma INR	1.85 (1.20–>28)	2.04 (1.26–3.64)	2.00 (1.21–3.41)

**Table 3:** Agreement of POCT INR with plasma INR measurements. Kappa value was 0.810 (95% CI: 0.732–0.889).

		POCT INR			Total
		INR < 2	INR 2–3	INR > 3	
Plasma INR	INR < 2	47	8	0	55
	INR 2–3	4	26	8	38
	INR > 3	0	1	11	12
Total		51	35	19	105

## Data Availability

The data used to support the findings of this study are available on request via mail to manu.henckes@gza.be.
